# Metabolic Regulation as a Consequence of Anaerobic 5-Methylthioadenosine Recycling in *Rhodospirillum rubrum*

**DOI:** 10.1128/mBio.00855-16

**Published:** 2016-07-12

**Authors:** Justin A. North, Jaya Sriram, Karuna Chourey, Christopher D. Ecker, Ritin Sharma, John A. Wildenthal, Robert L. Hettich, F. Robert Tabita

**Affiliations:** a Department of Microbiology, The Ohio State University, Columbus, Ohio, USA; b Chemical Sciences Division, Oak Ridge National Laboratory, Oak Ridge, Tennessee, USA; c University of Tennessee-ORNL Graduate School of Genome Science and Technology, Knoxville, Tennessee, USA

## Abstract

Rhodospirillum rubrum possesses a novel oxygen-independent, aerobic methionine salvage pathway (MSP) for recycling methionine from 5-methylthioadenosine (MTA), the MTA-isoprenoid shunt. This organism can also metabolize MTA as a sulfur source under anaerobic conditions, suggesting that the MTA-isoprenoid shunt may also function anaerobically as well. In this study, deep proteomics profiling, directed metabolite analysis, and reverse transcriptase quantitative PCR (RT-qPCR) revealed metabolic changes in response to anaerobic growth on MTA versus sulfate as sole sulfur source. The abundance of protein levels associated with methionine transport, cell motility, and chemotaxis increased in the presence of MTA over that in the presence of sulfate. Purine salvage from MTA resulted primarily in hypoxanthine accumulation and a decrease in protein levels involved in GMP-to-AMP conversion to balance purine pools. Acyl coenzyme A (acyl-CoA) metabolic protein levels for lipid metabolism were lower in abundance, whereas poly-β-hydroxybutyrate synthesis and storage were increased nearly 10-fold. The known *R. rubrum* aerobic MSP was also shown to be upregulated, to function anaerobically, and to recycle MTA. This suggested that other organisms with gene homologues for the MTA-isoprenoid shunt may also possess a functioning anaerobic MSP. In support of our previous findings that ribulose-1,5-carboxylase/oxygenase (RubisCO) is required for an apparently purely anaerobic MSP, RubisCO transcript and protein levels both increased in abundance by over 10-fold in cells grown anaerobically on MTA over those in cells grown on sulfate, resulting in increased intracellular RubisCO activity. These results reveal for the first time global metabolic responses as a consequence of anaerobic MTA metabolism compared to using sulfate as the sulfur source.

## IMPORTANCE

In nearly all organisms, sulfur-containing byproducts result from many metabolic reactions. Unless these compounds are further metabolized, valuable organic sulfur is lost and can become limiting. To regenerate the sulfur-containing amino acid methionine, organisms typically employ one of several variations of a “universal” methionine salvage pathway (MSP). A common aspect of the universal MSP is a final oxygenation step. This work establishes that the metabolically versatile bacterium *Rhodospirillum rubrum* employs a novel MSP that does not require oxygen under either aerobic or anaerobic conditions. There is also a separate, dedicated anaerobic MTA metabolic route in *R. rubrum*. This work reveals global changes in cellular metabolism in response to anaerobic MTA metabolism compared to using sulfate as a sulfur source. We found that cell mobility and transport were enhanced, along with lipid, nucleotide, and carbohydrate metabolism, when cells were grown in the presence of MTA.

## INTRODUCTION

**FIG 1:**
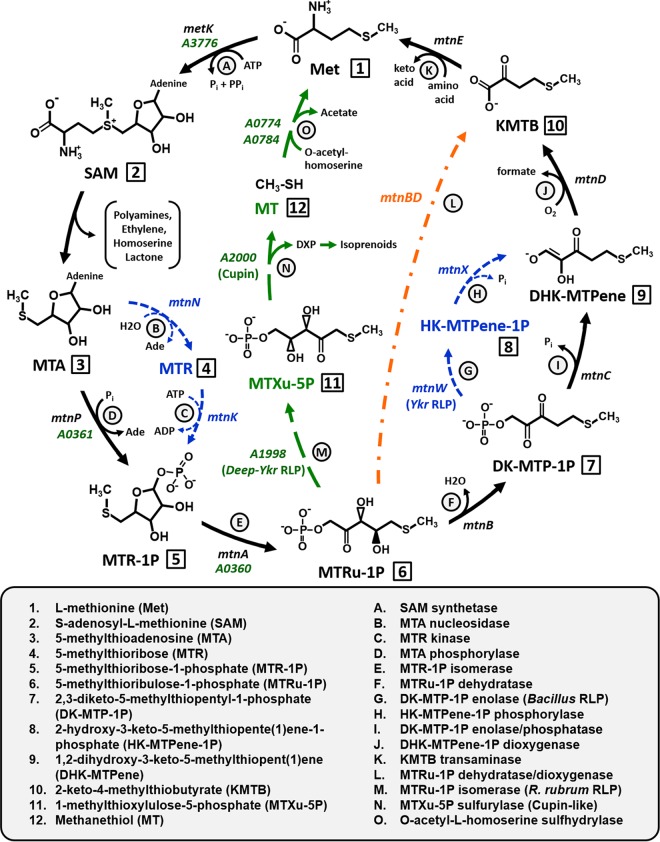
Variations on aerobic methionine salvage pathway. *K. pneumoniae* is shown with black arrows as a reference, *B. subtilis* variations are shown in blue, the *R. rubrum* MTA-isoprenoid shunt is shown in green, and the *Tetrahymena* sp. *mtnBD* fusion gene is shown in orange. Gene designations are given in italics for each enzyme (letters) in the pathways; numbers correspond to pathway compounds.

5-Methylthioadenosine (MTA) is a critical metabolic byproduct resulting from multiple essential cellular processes, including polyamine synthesis for cell proliferation, homoserine lactone production in quorum sensing, and ethylene hormone production for fruit ripening in plants (1–3). The inability to further metabolize MTA results in a reduction of available sulfur pools, and intracellular buildup of MTA can lead to cytotoxicity (4–6). In a wide variety of organisms, from microbes to plants and animals, MTA is detoxified and converted into usable l-methionine by the “universal” methionine salvage pathway (MSP) (Fig. 1, black arrows) (2). The importance of a functional MSP is underscored by the fact that many cancers develop a defective MSP, wherein the first gene of the universal MSP, MTA phosphorylase (Fig. 1, letter D), is deleted or downregulated, leading to an intercellular buildup of MTA that facilitates carcinoma aggression (7–9).

In the universal MSP, MTA is recycled to methionine by six consecutive reactions: nucleosidase/phosphorylase, isomerase, dehydratase, enolase/phosphatase, dioxygenase, and transamination (Fig. 1, letters D to F and I to K). However, recent studies have revealed a mechanistic diversity for MTA metabolism in the microbial community, resulting in variations on the universal MSP theme (10–12). In *Bacillus subtilis*, the MTA phosphorylase is replaced by a separate MTA nucleosidase and 5-methylthioribose (MTR) kinase (Fig. 1, letters B and C) (13, 14). Additionally, the bifunctional 2,3-diketo-5-methylthiopentyl-1-phosphate (DK-MTP-1P) enolase/phosphatase (Fig. 1, letter I) is replaced by a DK-MTP-1P enolase and subsequent 2-hydroxy-3-keto-5-methylthiopente(1)ene-1-phosphate (HK-MTPene-1P) phosphatase (Fig. 1, letters G and H) (15, 16). In *Tetrahymena* sp., a multifunctional fusion enzyme catalyzes the dehydratase, enolase/phosphatase, and dioxygenase reactions to generate 2-keto-4-methylthiobutyrate (KMTB) from 5-methylthioribulose-1-phosphate (MTRu-1P) (Fig. 1, letter L) (11). Some of these variations employ a RubisCO-like protein (RLP) (16–21). In *B. subtilis*, a Ykr class RLP (Fig. 1, letter G, *mtnW*) functions as the DK-MTP-1P enolase. RLPs, or form IV RubisCOs, have high structural similarity to bona fide forms of RubisCO, which carry out carboxylation/oxygenation of ribulose-1,5-bisphosphate (RuBP). However, key active site substitutions have left RLPs incapable of catalyzing RubisCO-dependent carboxylation or oxygenation (22–25).

The mechanistic variations described thus far are ultimately oxygen dependent due to the requirement of the dioxygenase (Fig. 1, letter J). Indeed, under anaerobic conditions, MTA metabolism is not supported in eukaryotes, *B. subtilis*, *Klebsiella pneumoniae*, *Escherichia coli*, and many facultative anaerobes (2). Recently, the first aerobic MSP that does not employ a dioxygenase to metabolize MTA was described in *Rhodospirillum rubrum*. Rather, a novel MTA-isoprenoid shunt links MTA metabolism to methanethiol (MT) release for methionine regeneration and 1-deoxyxylulose-5-phosphate (DXP) synthesis for isoprenoid metabolism (Fig. 1, green arrows) (12). This is performed by a Deep-Ykr class RLP, which catalyzes the isomerization of MTRu-1P to 1-methylthioxylulose-5-phosphate (MTXu-5P) (Fig. 1, letter M, Rru_A1998) (12, 17). MTXu-5P is subsequently metabolized by a cupin-type MTXu-5P sulfurylase to generate MT and DXP (Fig. 1, letter N, Rru_A2000). Finally, MT as a glutathione adduct is coupled with *O*-acetylhomoserine by *O*-acetylhomoserine sulfhydrylase to regenerate methionine (Fig. 1, letter O, Rru_A0774 and Rru_A0784) (12, 26, 27). Moreover, this same oxygen-independent MTA-isoprenoid shunt appears to also function under anaerobic conditions in *R. rubrum*, along with an apparently separate, purely anaerobic MTA route involving RubisCO (28), representing the first potential anaerobic MSPs described in any organism.

**FIG 2:**
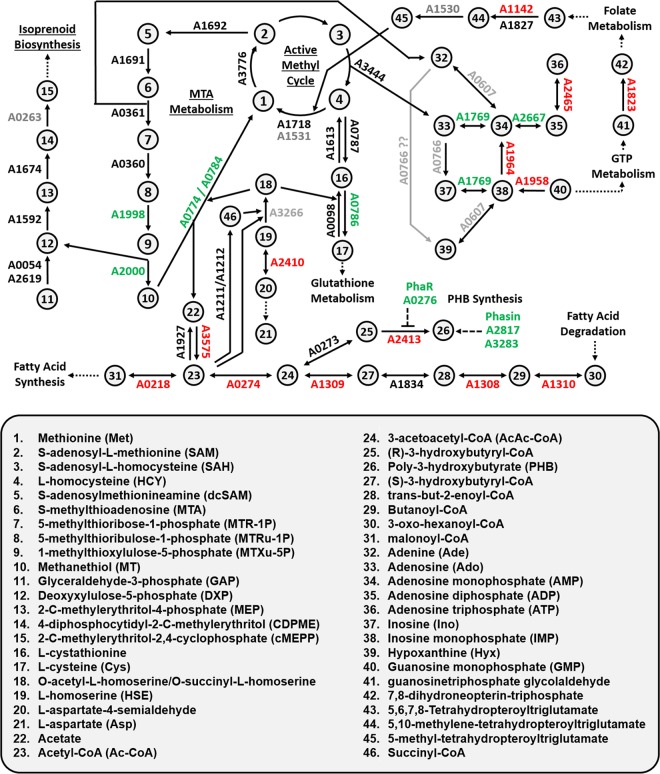
Map of metabolic pathways regulated by anaerobic growth on MTA compared to sulfate observed by deep proteomics. Gene designations are given beside each reaction. Gray, protein not identified; black, protein identified; green, protein increased in abundance; red, protein decreased in abundance. The name of each numbered chemical is listed below the figure.

In the current study, we employed a combination of deep proteomics profiling, reverse transcriptase quantitative PCR (RT-qPCR), and directed metabolite detection to examine metabolic changes resulting from anaerobic growth in the presence of MTA instead of sulfate as sole sulfur source in *R. rubrum* (Fig. 2). We found that numerous proteins associated with cell motility and methionine transport increased in abundance. *De novo* purine biosynthesis proteins decreased in abundance due to purine salvage of adenine from MTA. There was also a decrease in numerous acyl coenzyme A (acyl-CoA) metabolic enzyme levels involved in fatty acid metabolism, whereas poly-β-hydroxybutyrate (PHB) synthesis and phasin protein synthesis for PHB granule storage increased nearly 10-fold. Expression of *cbbM* transcript and RubisCO protein levels both increased by over 10-fold, resulting in a 2-fold increase in *in vivo* RubisCO carboxylase activity, consistent with the requirement for RubisCO during anaerobic MTA metabolism (21, 28). Last, during anaerobic growth on MTA, we observed an increased abundance of proteins known to function in the *R. rubrum* aerobic MSP (12, 27), with these genes also appearing to be transcriptionally upregulated in the presence of MTA. Subsequent knockout strain analysis and directed metabolite detection under anaerobic growth conditions revealed the same metabolites being involved in the *R. rubrum* aerobic MSP. This established that the *R. rubrum* MTA-isoprenoid shunt was functional for both aerobic and anaerobic MTA metabolism and, based on gene sequence homology, suggested that other organisms may possess a functioning MTA-isoprenoid shunt for anaerobic MSP. Moreover, these results reveal that *R. rubrum* metabolism is globally regulated under anaerobic conditions to support growth on MTA.

## RESULTS

### Global proteome response to anaerobic growth on MTA versus sulfate.

To determine the cellular pathways affected as a consequence of growth on MTA versus sulfate, we compared the proteome profiles of *R. rubrum* cultures grown in the presence of MTA and those grown in the presence of sulfate as sole sulfur source (Table 1; see also Table S1 in the supplemental material). *R. rubrum* contains a 4,352,825-bp chromosome and a 53,732-bp megaplasmid with the total possibility of 3,838 protein-coding genes and 83 RNA genes (29). A total of 1,501 proteins were observed by proteomic measurements across all data sets, representing 38.5% of the genome. Of these, 326 proteins showed changes in abundance levels in response to anaerobic growth on MTA versus sulfate as sulfur source (Table 1; see also Table S2 in the supplemental material). A majority of the observed proteins (929) were detected in both cells grown on MTA and cells grown on sulfate, while 304 proteins were observed only in MTA-grown cells, and 268 proteins were observed only in sulfate-grown cells (see Fig. S1A and Table S2).

**TABLE 1:** Proteins regulated by growth on MTA^a^

Gene identifier	Protein name	Fold change
A0274	Acetyl-CoA *C*-acetyltransferase	−2.30
A0276	PHB synthesis repressor, PhaR	1.69
A0774	*O*-Acetylhomoserine sulfhydrylase	3.14
A0779	MetQ-like solute-binding protein	6.32
A0784	*O*-Acetylhomoserine sulfhydrylase	3.88
A0786	Cystathionine gamma-synthase	10.88
A0788	MetN ABC transporter	9.44
A0791	NlpA^b^ lipoprotein	6.38
A1142	Glycine hydroxymethyltransferase	−3.09
A1308	Acyl-CoA dehydrogenase	−11.41
A1309	Acetoacetyl-CoA reductase	−4.92
A1310	Acetyl-CoA *C*-acyltransferase	−4.38
A1334	Ribose-5-phosphate isomerase *B*	9.82
A1823	3,4-Dihydroxy-2-butanone-4P synthase	−1.80
A1958	GMP reductase	−12.04
A1964	Adenylosuccinate synthase	−12.18
A1998	RubisCO-like MTRu-1P isomerase	8.13
A2000	Cupin-like MTXu-5P sulfurylase	0.96
A2400	RubisCO, form II	10.57
A2404	Phosphoribulokinase	9.50
A2413	PHB synthase, class I	−3.61
A2417	MetQ periplasmic solute-binding protein	2.63
A2419	MetN ABC transporter	2.28
A2465	Pyruvate kinase	−1.27
A2817	Phasin	14.52
A3206	CheY response regulator protein	8.83
A3283	Phasin-like activator of PHB degradation	1.51
A3575	Acetate-CoA ligase	−2.33

a Protein name and gene correspond to annotated *R. rubrum* ATCC 11170 genome (NCBI reference sequence NC_007643.1). Fold change is the increase (+) or decrease (−) in protein abundance observed in MTA- versus sulfate-grown cells by deep proteomics.

b NlpA, inner membrane lipoprotein A.

To verify consistency in sample preparation between the two different growth conditions, we analyzed the cellular localization patterns of the identified proteins using PSORT v3.0, a subcellular localization prediction tool (30). Although the total number of proteins identified in MTA- versus sulfate-grown cells differed, the distribution patterns of proteins per their predicted cellular locations were similar for the two groups (see Fig. S1B in the supplemental material). These data point toward efficient cellular lysis and protein extraction from cells grown under each condition.

To visualize global proteome changes, the proteins identified from MTA- and sulfate-grown cells were compared based on the functional category distribution of the identified proteins (see Fig. S1C in the supplemental material). In MTA-grown cells, proteins involved in cell maintenance and repair, including transcription (category K); DNA replication, recombination, and repair (category L); cell wall and membrane synthesis (category M); signal transduction (category T); and defense mechanisms (category V), along with several proteins of unknown function, were more abundant than those in sulfate-grown cells. Conversely, proteins involved in cell growth and proliferation, including energy production and conversion (category C); cell cycle control and division (category D); amino acid (category E), nucleotide (category F), and lipid (category I) transport; and secondary metabolite biosynthesis (category Q) were more abundant in sulfate-grown cells. These differences in proteome profiles in cells grown anaerobically on MTA compared with those grown on sulfate represent an observable shift in global metabolism as a result of growth on MTA instead of sulfate as sole sulfur source.

### Methionine transport system.

Methionine transport in many organisms is attributed to members of the MetINQ complex. This complex consists of an ATP-binding cassette transporter (MetN), permease (MetI), and periplasmic solute-binding protein (MetQ) to facilitate the transport of methionine across the cell membrane at the expense of ATP (31, 32). *R. rubrum* homologous methionine transport proteins were analyzed by STRING v10 analysis (33) to identify interacting partners forming a putative MetINQ complex (see Fig. S2 in the supplemental material). Both *R. rubrum* orthologues of MetN (Rru_0788 and Rru_A2419) increased in response to growth on MTA versus sulfate (Table 1). Additionally, one of the two MetQ orthologues (Rru_A2417), another MetQ-like periplasmic substrate binding protein (Rru_A0779), and an annotated inner membrane lipoprotein (NlpA, Rru_A0791), similar to MetQ in other organisms, were also observed in increased abundance.

### Chemotaxis is essential for growth on MTA.

**FIG 3:**
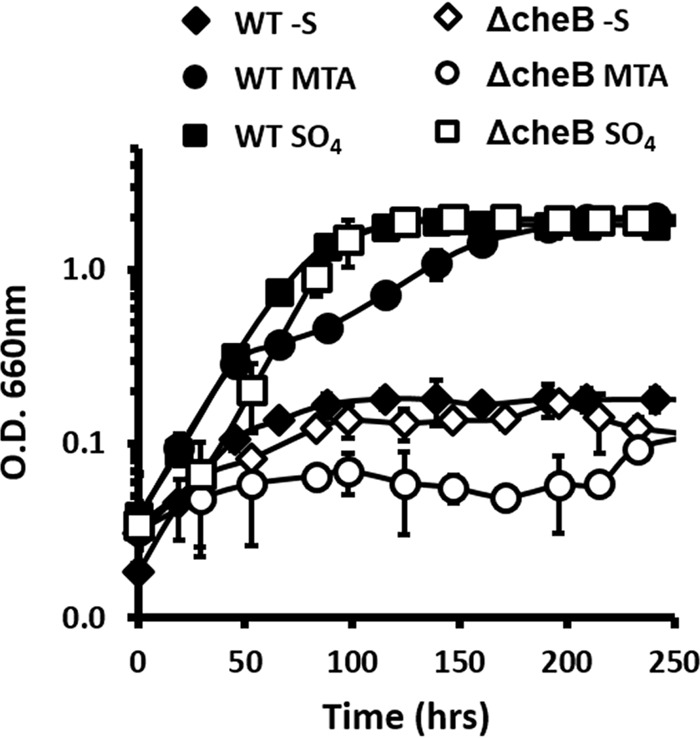
Anaerobic growth of *R. rubrum* wild-type (WT) strain (black) and transposon mutant with disrupted CheB methylesterase gene (ΔRru_A1406; see Fig. S4 in the supplemental material) (white) in malate minimal medium without sulfur source (diamonds) or supplemented with 500 µM MTA (circles) or 500 µM sulfate (squares). Error bars represent standard deviations for *n* = 3 independent growth experiments.

The role of cellular chemotaxis in the presence of MTA is underpinned by a 10-fold increase in abundance of the motor switch control protein CheY (Rru_A3206) (Table 1) and is further corroborated by an observed increase in multiple proteins of the flagellar motor complex, including FliG and FliN (see Fig. S3 in the supplemental material). Binding of phosphorylated CheY to FliN of the C-ring switches flagellar rotation from the default counterclockwise rotation (forward swimming) to clockwise rotation (tumbling motion) (34). Repellants, such as MTA, that bind to the methyl-accepting chemotaxis proteins (MCPs) result in increased MCP phosphorylase activity. Activated MCP phosphorylates CheY, which in turn increases the flagellar rotor switching frequency from forward to tumbling swimming to avoid the repellant (35, 36). Requirement for proper chemotaxis in the presence of MTA is bolstered by an *R. rubrum* strain bearing a Tn*5* disruption of the CheB (Rru_A1406) gene (Fig. 3; see also Fig. S3). This mutant is capable of growing on sulfate but not MTA as a sulfur source. Increased MCP phosphorylase activity in the presence of repellants results in phosphorylated CheB, which in turn demethylates the MCP to reduce MCP sensitivity and thus phosphorylase activity in response to the repellant (37, 38). This functions to prevent unproductive, continuous tumbling motion. In *B. subtilis*, deletion of CheB resulted in the inability to properly respond and swim in the presence of repellants (39). Collectively, these results point to a specific cellular chemotaxis response in the presence of MTA.

### Acylhomoserine and PHB metabolism.

During *R. rubrum* anaerobic growth on MTA versus sulfate, the levels of numerous enzymes utilizing acetyl-CoA as a substrate were observed in lower abundance (Fig. 2; Table 1) (23). However, abundance levels were not affected in either acetyl-CoA hydrolase (Rru_A1927) or succinyl-CoA synthetase (Rru_A1211/Rru_A1212), which regulate acetyl-CoA and succinyl-CoA pools, respectively (Fig. 2; see also Table S2 in the supplemental material) (23, 40). Furthermore, our previous *in vitro* characterization of the *R. rubrum* O-acetylhomoserine sulfhydrylase (Rru_A0774) showed similar reaction kinetics using *O*-acetylhomoserine (*k*_cat_ = 3.5 s^−1^) and *O*-succinylhomoserine (*k*_cat_ = 1.3 s^−1^) (12). This is consistent with the conclusion that *O*-acetylhomoserine and *O*-succinylhomoserine pools are important for anaerobic MTA metabolism (Fig. 2) (18). Surprisingly, the *R. rubrum* MetX (Rru_A3266) orthologue, which catalyzes the synthesis of *O*-acetylhomoserine from acetyl-CoA and serine, was not observed by deep proteomics. Additionally, *R. rubrum* does not appear to possess a MetA orthologue (EC 2.3.1.46, homoserine *O*-succinyltransferase) for synthesis of *O*-succinylhomoserine (29).

The regulation of other acyl-CoA pools during anaerobic growth on MTA was further implied by decreased abundance of fatty acid degradation and poly-3-hydroxybutyrate (PHB) biosynthesis protein levels (Fig. 2; Table 1). The observed 4-fold decrease in PHB synthase levels (Rru_A2413) apparently results in part from a 1.7-fold increase in the PHA operon repressor, PhaR (Rru_A0276). Surprisingly, the most distinctively abundant protein (14.5-fold) in MTA- versus sulfate-fed cells was a phasin (Rru_2817) from the phasin 2 family, which includes *Ralstonia eutropha* PhaP1 (41). This increase in phasin protein levels correlated with a 45- ± 19-fold increase in mRNA transcript, as determined by RT-qPCR. In addition, the phasin-like activator of PHB degradation (ApdA, Rru_A3283) (42) slightly increased (1.5-fold). PHB granules are formed primarily under anaerobic conditions for carbon and energy storage, and phasin expression is observed to coincide with PHB accumulation (43, 44). In *R. rubrum* and other organisms, phasins interact with PHB granules to regulate granule synthesis, morphology, and coalescence and to prevent nonspecific protein binding (44, 45).

**FIG 4:**
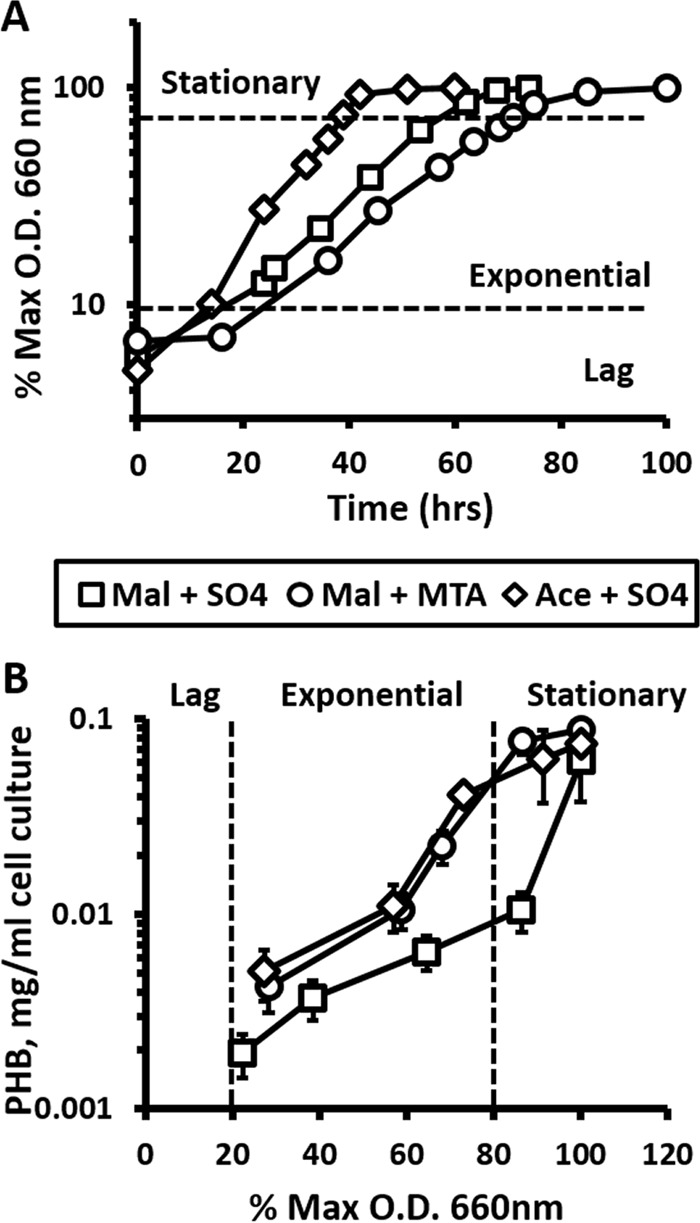
PHB production in response to MTA. (A) Growth of *R. rubrum* wild-type strain grown anaerobically in the presence of malate and sulfate (*T* = 19.5 h, squares), malate and MTA (*T* = 23 h, circles), and acetate and sulfate (*T* = 12.9 h, diamonds) as carbon and sulfur sources, respectively. Growth data are plotted as percentages of the maximum optical density at 660 nm (% Max O.D. 660 nm) reached by the culture. (B) Cellular PHB levels during growth of *R. rubrum* wild-type strain in panel A at the given culture optical density at 660 nm. Error bars are the standard deviations from 3 independent experiments.

In order to determine how PHB accumulation is affected as a consequence of growth on MTA as sole sulfur source, we quantified PHB levels in cells grown on acetate or malate as a carbon source and sulfate or MTA as a sulfur source (Fig. 4). Malate-grown cells with MTA as a sulfur source accumulated 3- to 7-fold more PHB than did those with sulfate during exponential growth. Moreover, cells grown on malate and MTA produced PHB at the same rate as did cells grown on acetate and sulfate, the latter of which is known to stimulate PHB production (42). This increase in PHB production in the presence of MTA correlates with the observed increase in both phasin protein and transcript levels. Clearly, even though PHB synthase (Rru_A2413) levels decrease in the presence of MTA, there is an increased flux of (*R*)-3-hydroxybutyryl-CoA toward PHB synthesis.

### Purine salvage from MTA.

During aerobic MTA metabolism, the first enzymatic step hydrolyzes MTA to produce MTR-1P and adenine (12). Presumably, adenine is salvaged to generate other nucleosides and nucleotides for cellular metabolism. This is substantiated by the observation that the GMP-to-AMP conversion pathway, which balances A and G levels in the cell, is strongly downregulated. GMP reductase (Rru_A1958), which irreversibly converts GMP to IMP, and adenylosuccinate synthase (Rru_A1964), which generates AMP from IMP, each show a 12-fold decrease in protein levels (Fig. 2; Table 1). This is evidently in response to the excess adenine flux from MTA metabolism.

**FIG 5:**
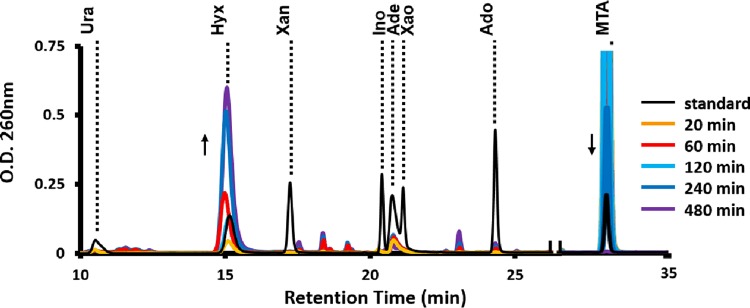
HPLC identification of purines resulting from MTA metabolism. The *R. rubrum* wild-type strain was grown anaerobically in the presence of sulfate and then fed with MTA for the indicated amount of time (minutes) before resolution of metabolites via 260-nm absorbance present in the medium after reverse-phase liquid chromatography. “Standard” (black line) is the retention time of known purine base and nucleoside standards (see Fig. S4A in the supplemental material). Ura, urate; Hyx, hypoxanthine; Xan, xanthine; Ino, inosine; Ade, adenine; Xao, xanthosine; Ado, adenosine; MTA, 5-methylthioadenosine.

To further assess purine salvage, we performed reversed-phase high-performance liquid chromatography (HPLC) analysis of purine bases and nucleosides produced by *R. rubrum* during anaerobic MTA metabolism versus growth on sulfate. When the organism was fed with sulfate as sole sulfur source, no accumulation of any purine species was observed (data not shown). However, when the organism was fed with MTA, there was a concomitant accumulation of primarily hypoxanthine along with adenine and adenosine based on known standards (Fig. 5). The observation of adenine versus nucleosides with similar retention times was further confirmed by collection and subsequent electrospray ionization mass spectroscopy (see Fig. S5C in the supplemental material; C_5_H_6_N_5_^+^, *m/z* = 136.0624, δ = 4.5 ppm). Hypoxanthine was confirmed by specific enzyme assay via conversion to urate by xanthine oxidase (see Fig. S4A and B). Furthermore, the absence of xanthine and urate in MTA-fed cells is consistent with the observation that *R. rubrum* lacks any homologue of xanthine oxidase (EC 1.17.1.4) (29).

### MTA leads to increased RubisCO gene expression, protein abundance, and enzymatic activity.

**FIG 6:**
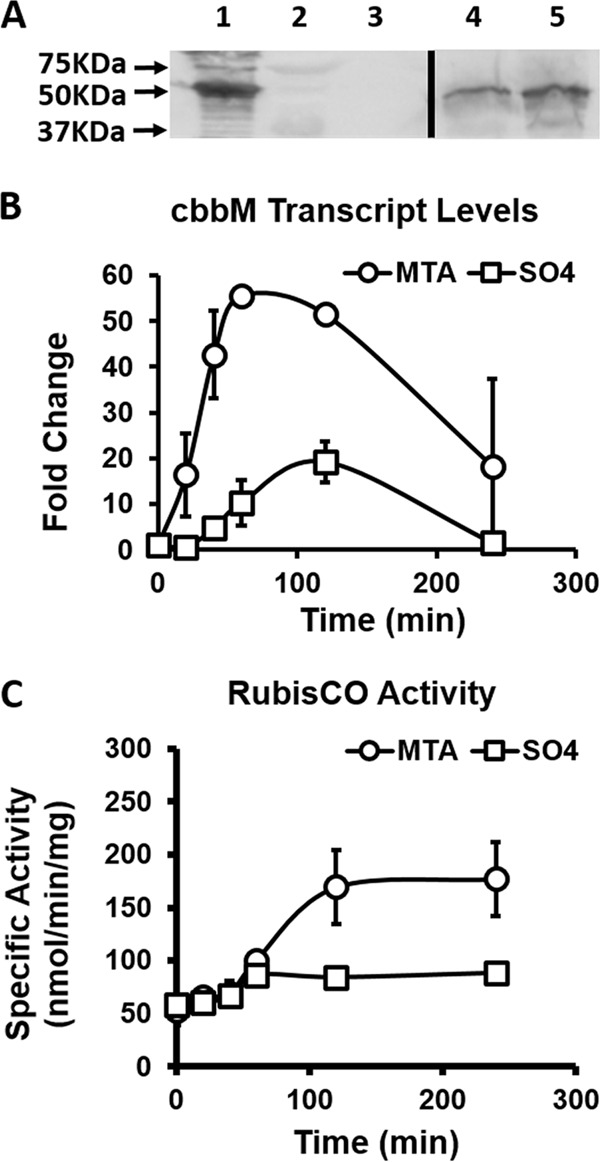
RubisCO regulation by MTA. (A) Immunoblotting analysis for RubisCO proteins. Lane 1, purified *R. rubrum* RubisCO; lane 2, molecular mass standards; lane 3, RubisCO deletion strain (I19A) grown anaerobically on sulfate; lane 4, wild-type strain grown anaerobically on sulfate; lane 5, wild-type strain grown anaerobically on MTA. (B) RT-qPCR of RubisCO gene (Rru_A2400) levels in wild-type strain fed anaerobically with MTA (circles) or sulfate (squares). (C) RubisCO specific carboxylase activity in nanomoles of CO_2_ fixed per minute per milligram of total cell protein in wild-type strain fed anaerobically with MTA (circles) or sulfate (squares). Error bars represent the standard deviations for *n* = 3 independent feeding experiments.

Detailed proteomics interrogation revealed that RubisCO protein (Rru_2400) levels increased nearly 11-fold in cells grown anaerobically on MTA over those in cells grown on sulfate. Additionally, the two preceding enzymes of the carbon fixation pathway, ribose-5-phosphate isomerase (RPI; Rru_1334) and phosphoribulokinase (PRK; Rru_2404), also increased nearly 10-fold each (Table 1). Immunoblotting analysis confirmed that RubisCO levels were elevated during anaerobic MTA growth (Fig. 6A), while no RubisCO was observed in the *R. rubrum* cbbM (RubisCO) deletion strain (I19A, ΔRru_A2400), which cannot grow on MTA as sole sulfur source (21).

To determine how MTA regulates RubisCO gene expression and activity, we performed MTA feeding experiments and followed the temporal evolution of RubisCO transcript by RT-qPCR and RubisCO activity by carboxylase assay (46). Cells were grown anaerobically to mid-exponential phase and then washed under sulfur-depleted conditions and fed with either MTA or sulfate. At the point of feeding sulfate or MTA, transcript and RubisCO activity levels were identical in the two populations (Fig. 6B and C). During the first 60 min postfeeding, there was an ~5-fold increase in RubisCO transcript levels in MTA-fed cells over those in sulfate-fed cells, but the levels then returned to initial levels at later times (Fig. 6B). This spike in transcript and concomitant protein levels resulted in RubisCO activity levels increasing by 2- to 3-fold (Fig. 6C). Clearly, MTA metabolism upregulates RubisCO expression, resulting in increased cellular RubisCO activity. This further underpins the requirement for RubisCO during anaerobic MTA metabolism (21, 28).

### Methionine salvage pathway under anaerobic growth conditions.

Previous studies of *R. rubrum* aerobic MTA metabolism by metabolomics and transcriptomics revealed that its entire aerobic MSP (Fig. 1, green arrows) was upregulated during growth on MTA versus sulfate (27). Here, while the same set of enzymes was observed by proteomics under anaerobic growth on both sulfate and MTA (see Table S2 in the supplemental material), only the *R. rubrum* RLP (Fig. 2; Rru_A1998), cupin (Fig. 2; Rru_A2000), and *O*-acetylhomoserine sulfhydrylase (Fig. 2; Rru_A0774/0784) showed changes in protein levels in response to growth on MTA versus sulfate (Table 1). The first two enzymes of the known aerobic MSP, MTA phosphorylase (Fig. 1, letter D, and Fig. 2; Rru_A0361) and MTR-1P isomerase (Fig. 1, letter E, and Fig. 2; Rru_A0360), were measured at similar levels in cells grown anaerobically on MTA and on sulfate. To further identify potential involvement and regulation of these enzymes in response to anaerobic MTA metabolism, we performed RT-qPCR on the MTA phosphorylase and RLP genes. Consistent with a lack of MTA phosphorylase regulation at the protein level, transcript levels did not change in cells grown on MTA compared with those grown on sulfate (threshold cycle [ΔΔ*C_T_*], 1.3 ± 0.4). This suggests that expression of MTA phosphorylase (Rru_A0361) and of the preceding gene, that for MTR-1P isomerase (Rru_A0360), is not regulated by MTA during anaerobic growth, whereas it is regulated by MTA under aerobic conditions (12, 27). However, there was a 7- ± 3-fold increase in RLP transcript levels in cells grown anaerobically on MTA over that in cells grown on sulfate, similar to aerobic growth. This shows that the RLP is regulated by MTA under both anaerobic and aerobic conditions.

**FIG 7:**
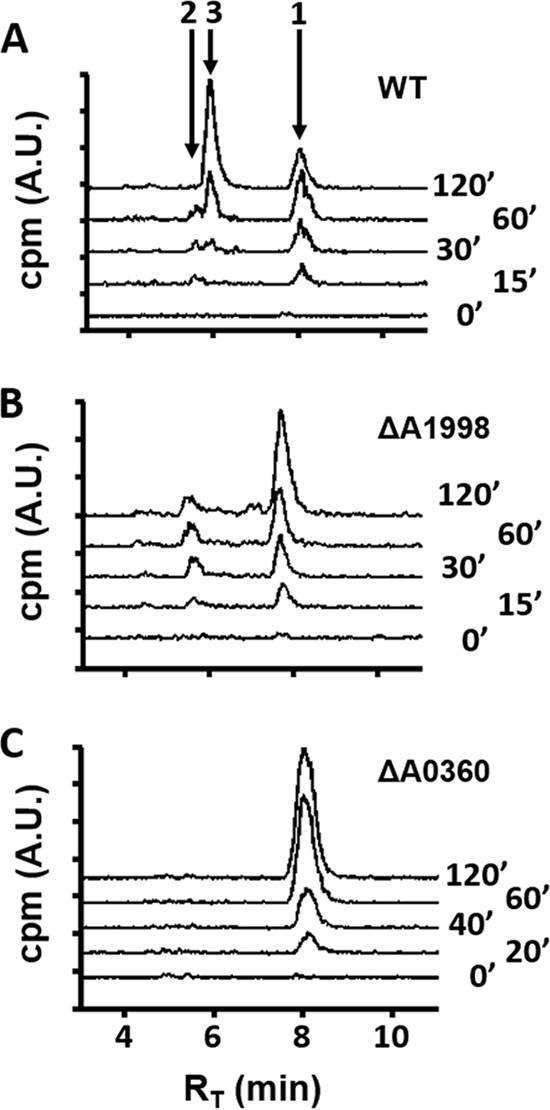
Reversed-phase separation and ^14^C radiometric detection of MTA-fed cells. *R. rubrum* wild type (WT) (A), RLP (MTRu-1P isomerase) deletion strain (ΔRru_A1998) (B), and MTR-1P isomerase deletion strain (ΔRru_A0360) (C) grown anaerobically on sulfate and then fed with [*methyl*-^14^C]MTA for the indicated time (minutes) before resolution of ^14^C-containing metabolites present in the medium by reversed-phase chromatography. Peak 1 (retention time [*R_T_*,], 8.2 min) is MTR-1P, peak 2 (*R_T_*, 5.6 min) is MTRu-1P, and peak 3 (*R_T_*, 5.8 min) is MTXu-5P, based on known standards (see Fig. S2A in the supplemental material).

Our recent metabolomics studies of *R. rubrum* anaerobic MTA metabolism showed increased levels of MTR-1P, MTRu-1P, MT, DXP, and subsequent isoprenoid precursors in cells fed with MTA over those in cells fed with sulfate as a sulfur source (28). This suggested that the known *R. rubrum* aerobic MSP (Fig. 1, green arrows) may also function during anaerobic growth. To determine if the MTA phosphorylase, MTR-1P isomerase, and RLP (Fig. 1, letters D, E, and M) are functionally involved in anaerobic MTA metabolism, we performed ^14^C radiochromatography by HPLC of knockout strains fed with [*methyl*-^14^C]MTA. In the wild-type strain, MTR-1P, MTRu-1P, and MTXu-5P/MTRu-5P (Fig. 7A; also see Fig. S5A in the supplemental material) and MT (28) levels increased upon feeding cells anaerobically with MTA. Inactivation of the *R. rubrum* RLP resulted in loss of MTXu-5P/MTRu-5P (Fig. 7B) and concomitant loss of MT generation (28). When the MTR-1P isomerase was inactivated, only MTR-1P was observed (Fig. 7C). Coordinately, when the MTA phosphorylase was inactivated, MTR-1P was no longer observed as well (see Fig. S5B). Furthermore, production of each metabolite is specific to a single corresponding gene. Previously, under aerobic conditions, it was demonstrated that complementation of each knockout strain with the corresponding protein restored production of the specific metabolite (12). Taken together, these results establish that the *R. rubrum* aerobic MSP (MTA-isoprenoid shunt) coordinately functions anaerobically and that it is transcriptionally upregulated via the RLP during growth on MTA.

## DISCUSSION

Recycling MTA, a dead-end byproduct from polyamine, homoserine lactone, and ethylene biosynthesis, is necessary for most organisms to maintain usable sulfur pools in a low-sulfur environment and to prevent MTA cytotoxicity. In eukaryotes and many prokaryotes, MTA is recycled into usable methionine by the universal methionine salvage pathway, which requires oxygen for the final metabolic step to occur (Fig. 1, black arrows). Many anaerobic organisms cannot metabolize MTA into usable sulfur, presumably due to the oxygen requirement of the universal MSP, or due to the lack of an MSP altogether (2). However, we have observed that *Rhodospirillum rubrum* (21) and *Rhodopseudomonas palustris* (40) are able to metabolize MTA anaerobically, indicating the existence of an anaerobic MSP(s). Through deep proteomics, qRT-PCR, and directed metabolite detection, we have observed that anaerobic growth on MTA instead of sulfate as the sulfur source leads to a global shift in metabolism (see Fig. S1C in the supplemental material). Of particular relevance are changes in pathways for (i) methionine transport, (ii) chemotaxis and the flagellar assembly, (iii) acyl-CoA and PHB metabolism, (iv) purine salvage, (v) RubisCO gene expression and protein synthesis, and (vi) MTA metabolism when cells are grown in the presence of MTA versus sulfate.

### Methionine transport.

The observed increase in protein levels of the methionine (MetINQ) could arise for at least two reasons: available methionine could be limiting in cells grown on MTA, or the methionine transport complex is involved in MTA uptake. In *Streptococcus mutans*, expression of the methionine uptake complex is upregulated when methionine is depleted or there is an excess of methionine intermediates such as homocysteine (47). Given that MTA is inhibitory to numerous enzymes (1–6) and that the MTA metabolism pathway is relatively inefficient catalytically as a sole source for methionine (1, 12), increased MetINQ levels may be an attempt to scavenge methionine. Alternatively, the MetINQ complexes are known to exhibit substrate variability. In *Nostoc muscorum* and *Bacillus subtilis*, a common MetINQ complex transports methionine, methionine-sulfoxide, methionine-sulfoxamine, and phosphinothricin (48, 49). Given that MTA is transported into *R. rubrum* as observed by metabolomics (28) and by [*methyl*-^14^C]MTA radiometric measurements (data not shown), it could be that the MetINQ complex is also responsible for MTA uptake. It will be of interest to determine if this upregulation of the methionine transport complexes serves to facilitate MTA uptake or an attempt to scavenge methionine.

### Chemotaxis.

The increase in cellular chemotaxis machinery, including flagellar motor and chemosensory components, is also consistent with a cellular response to scavenge methionine when MTA is present as sole sulfur source. Alternatively, given that MTA is cytotoxic to *R. rubrum* at millimolar levels (28) and constitutes a repellant as evidenced by elevated CheY levels (Table 1; also see Fig. S3 in the supplemental material) and the MTA growth-deficient CheB mutant (Fig. 3), these changes may be a chemotactic response due to the presence and uptake of MTA. Regardless, these two models are not mutually exclusive, and further experiments are required to determine the respective contributions from methionine depletion and the presence of MTA.

### Acyl-CoA and PHB metabolism.

Synthesis of PHB in *R. rubrum* and other bacteria requires acetyl-CoA and NADH. Furthermore, PHB production is stimulated under nutrient-limiting conditions, particularly low nitrogen and phosphorus, or when the carbon supply is high (42, 50). In a nutrient-limiting environment, cells store excess carbon and energy in the form of PHB until conditions become more favorable. In this study, available nutrients (ammonium, phosphorus, sulfate, or MTA) were supplied above limiting concentrations for *R. rubrum*. However, MTA as a sulfur source, via either toxicity or rate of metabolism to methionine, may be producing an effective sulfur-limited response. This is evidenced by the facts that *R. rubrum* grows 20 to 50% slower on MTA than on sulfate as sole sulfur source (Fig. 3) (28), numerous enzymes utilizing acetyl-CoA for lipid metabolism are in lower abundance (Fig. 2), and proteomic analysis shows a lower abundance of proteins involved in cell growth and proliferation, energy production and conversion, and cell cycle control and division (see Fig. S1C in the supplemental material) when grown on MTA than when grown on sulfate. Alternatively, the ribose moiety of MTA provides extra carbon that can be further metabolized to DXP and isoprenoids. This would allow carbon in the form of pyruvate and glycerol-3-phosphate normally used by DXP synthase to be channeled into PHB production. However, given that MTA is supplied at 0.5 mM and l-malate is supplied at 10 mM and that neither isozyme of DXP synthase (Rru_A0054 and Rru_A2619 [see Table S2 in the supplemental material]) changed in abundance in cells grown in the presence of MTA versus sulfate, it is unlikely that excess carbon flux from MTA produces a sufficient net carbon imbalance to result in the 3- to 7-fold increase in PHB accumulation. It will be of interest to determine if and how MTA regulates acetyl-CoA and other acyl-CoA pools to balance acetyl-/succinylhomoserine production for MTA metabolism, lipid/amino acid metabolism for cell growth, and PHB production for carbon storage.

### Purine salvage.

When metabolizing MTA, *R. rubrum* is faced with adenine and MTR-1P via MTA phosphorylase activity. In *R. rubrum*, adenine is further metabolized into hypoxanthine and adenosine, which are presumably salvaged for synthesis of other purines. For cells grown on MTA versus sulfate, this is supported by observed lower levels of GMP reductase (Rru_A1958) and adenylosuccinate synthase (Rru_A1964) (Table 1; Fig. 2), which convert GMP to AMP from *de novo* purine biosynthesis. Such was also observed for aerobic MTA metabolism in *R. rubrum* (27). Interestingly, in the related nonsulfur purple bacterium *Rhodopseudomonas palustris*, there appears to be no purine salvage from MTA, as only adenine accumulates and in stoichiometric amounts forms MTA (F. R. Tabita, unpublished observations). Surprisingly, based on homology, *R. rubrum* does not appear to possess many of the conventional purine salvage enzymes, particularly purine nucleosidase (EC 2.4.2.1), adenine deaminase (EC 3.5.4.2), and xanthine oxidase (EC 1.17.1.4) (29). As with many organisms, the *R. rubrum* MTA phosphorylase exhibits many similarities to other purine nucleosidases (2, 12). However, our previous studies with the *R. rubrum* enzyme indicate that the catalytic rate of hydrolysis for adenosine is ~30 times lower (<0.2 s^−1^) than for MTA and 5-deoxyadenosine (12). Moreover, neither the annotated *R. rubrum* orthologue of adenosine deaminase (Rru_A0766) nor that of purine phosphoribosyltransferase (Rru_A0607) was detected via proteomics. Further work is required to determine the precise purine salvage pathway in *R. rubrum*.

### Anaerobic MTA metabolism.

For metabolism of MTA to usable sulfur, proteomics and knockout metabolomics (Fig. 2 and 7) have confirmed our previous indications (28) that the *R. rubrum* MTA-isoprenoid shunt functions as an MSP anaerobically as well as aerobically. In this MSP, the RLP and cupin (Fig. 1, letters M and N) convert MTRu-1P into MT and DXP for methionine and isoprenoid synthesis, respectively, without the requirement for oxygen. Such an anaerobic MSP may be present in other bacteria (see Fig. S6 in the supplemental material), including *Rhodopseudomonas palustris*, *Meiothermus* sp., *Nitrococcus mobilis*, and *Halorhodospira halophila*, which possess homologues of the *R. rubrum* RLP and cupin in a similar gene organization.

Moreover, there is evidence for an additional anaerobic MSP as well. Our previous reports showed that while the *R. rubrum* RLP is not explicitly required to support anaerobic MTA metabolism, a bona fide RubisCO is, leading to production of methylthiolactate and *S*-methylcysteine (21, 28). These MTA-derived metabolites are not part of the MTA-isoprenoid shunt established as an anaerobic MSP in this study. RubisCO’s essential role in an anaerobic MSP is further underpinned by its 10-fold-increased abundance in transcript and protein levels and 2- to 3-fold increase in activity in response to MTA. One possible role of RubisCO in anaerobic MSP is direct participation in the metabolism of MTR-1P into *S*-methylcysteine or methylthiolactate. This is consistent with our observation that mutant RubisCOs devoid of carbon fixation activity are still able to support MTA metabolism in *R. rubrum* (28). An alternate model is that ribose-5-phosphate or ribulose-5-phosphate is generated during metabolism of MTR-1P into *S*-methylcysteine. This is supported by an observed 10-fold increase in RPI (Rru_1334) and PRK (Rru_2404), the latter of which converts ribose-5-phosphate to RuBP, RubisCO’s substrate. While RuBP buildup does lead to toxicity and slow growth in an *R. rubrum* RubisCO deletion strain (51), our previous studies indicate that toxic RuBP buildup is not the cause of the no-growth phenotype in RubisCO deletion strains grown anaerobically on MTA as sole sulfur source (28). Experiments are under way to isolate RubisCO’s specific role in anaerobic MTA metabolism and to elucidate additional steps in anaerobic MSPs.

## MATERIALS AND METHODS

### Bacterial strains.

The *R. rubrum* wild-type strain Str-2 is a spontaneous streptomycin-resistant derivative of type strain S1 (ATCC 11170) (29). The RubisCO deletion strain I19A (Δ*cbbM*) contains a kanamycin resistance gene inserted into the *cbbM* gene (Rru_A2400) (52). The MTA phosphorylase (Rru_A0361) and MTR-1P isomerase (Rru_A0360) genes were deleted as described in Text S1 in the supplemental material. The 5-methylthioribulose-1-phosphate isomerase gene (Rru_A1998) was deleted exactly as previously described (21).

### Growth conditions.

All *R. rubrum* strains were grown anaerobically at 30°C in sulfur-free Ormerod’s minimal medium using modifications as described previously (21) and supplemented with 20 mM sodium acetate (J. T. Baker) or 20 mM dl-malate (Sigma) and either 0.5 mM 5-methylthioadenosine (Sigma) or ammonium sulfate (Sigma). For growth, immunoblotting, RT-qPCR, PHB, and RubisCO activity analysis, photoheterotrophic cultures were grown in anaerobic test tubes under a nitrogen atmosphere. For proteomics analysis, cells were grown in 1-liter bottles fitted with anaerobic septa. All cultures were grown in duplicate.

### Tn*5* library screening.

A 2,000-colony partial mini-Tn*5* transposon library was created by conjugating *R. rubrum* with *E. coli* DH5α-λpir transformed with pRL27 (53) and screening for kanamycin-resistant *R. rubrum* transconjugants. Two-hundred-microliter photoheterotrophic cultures of each transconjugant were grown in 96-well plates under a 95:5 nitrogen-hydrogen atmosphere in thermally sealed bags (Scotchpak; 3M). Cultures were transferred by 48-pin stamp in an anaerobic chamber (Coy Laboratories) to 12% (wt/vol) sulfur-free solid agar plates prepared as described elsewhere (54) containing Ormerod’s minimal medium with either 500 µM MTA or ammonium sulfate. Plates were resealed in the same bag system and grown photoheterotrophically at 30°C to screen for MTA metabolic mutants. The location of the mini-Tn*5* insertion was isolated and sequenced as previously described (53).

### Protein purification and Western blot analysis.

The *R. rubrum* RubisCO, MTA phosphorylase, MTR-1P isomerase, and MTRu-1P isomerase (RLP) were purified as described previously (12). For immunoblotting analysis of RubisCO, purified protein and crude extracts were assayed as previously described (28). Immunoblots were developed with the Attophos (Amersham, Buckinghamshire, England) detection reagent according to the manufacturer’s instructions and visualized with a Molecular Dynamics Storm 840 imaging system.

### Feeding experiments for RubisCO transcript, carboxylase, and MTA metabolism studies.

The wild-type strain of *R. rubrum* was grown in the presence of sulfate to mid-exponential phase (optical density at 660 nm [OD_660_] of ~0.5), washed anaerobically three times with sulfur-free Ormerod’s minimal medium, and then resuspended to a final OD_600_ of ~0.3 in 10 ml Ormerod’s minimal medium with 0.25 mM MTA, [*methyl*-^14^C]MTA, or ammonium sulfate. Feedings proceeded anaerobically at 30°C for the designated amount of time, culture samples were centrifuged at 5,000 × *g* for 5 min at 20°C, and the spent medium and cell pellets were separately flash-frozen at −80°C for further analysis. Pellets for RT-qPCR were extracted as described below.

### PHB assay and RubisCO carboxylase activity.

Pellets for PHB quantification were resuspended in 10 ml of 5% sodium hypochlorite (Clorox) per 1-OD_660_ unit of original cell culture. PHB was extracted and quantified spectrophotometrically at 235 nm (Cary; Varian) according to the method of Law and Slepecky (55). Pellets for RubisCO carboxylase activity were resuspended in 0.5 ml 100 mM Bicine buffer (pH 8; Sigma), sonicated by a Branson Sonifier on ice for 3 min at a 50% duty cycle, and centrifuged at 15,000 × *g* at 4°C for 10 min to generate cell extract. RubisCO carboxylase activity was monitored by [^14^C]bicarbonate (PerkinElmer) carboxylation of ribulose-1,5-bisphosphate (Sigma) and was assayed as previously described (46).

### HPLC metabolite analysis.

Detection of *R. rubrum* metabolites due to MTA metabolism present in the spent medium was performed by reverse-phase HPLC on a Zorbax C_18_ (Agilent) column connected to a Shimadzu Prominence HPLC system with dual-wavelength detection (215 nm and 260 nm) and an inline β-RAM radiochromatography scintillation detector (IN/US Systems). Metabolites were eluted on a linear gradient from 0 to 50% acetonitrile (J. T. Baker) in 20 mM ammonium acetate over 30 min at 30°C. All purine base and nucleoside standards were from Sigma-Aldrich. Spent medium was treated with xanthine oxidase (Sigma) according to the manufacturer’s protocol. [*methyl*-^14^C]MTA was synthesized from *S*-[*methyl*-^14^C]adenosyl-l-methionine (PerkinElmer) by acid hydrolysis (56). Radiolabeled MTR-1P, MTRu-1P, and MTXu-5P standards were synthesized enzymatically from [*methyl*-^14^C]MTA using purified MTA phosphorylase, MTR-1P isomerase, and MTRu-1P isomerase as previously described (12).

### RNA extraction and real-time PCR.

Cells were harvested in exponential phase by centrifugation at 10,000 × *g* for 3 min and then resuspended in 1 ml of Tri reagent (Sigma-Aldrich; catalogue no. T9424). RNA was isolated as described previously (57). A reverse transcription reaction was performed by using Superscript reverse transcriptase (Invitrogen), and random hexamers were used as primers. Quantitative PCR was performed on a CFX96 real-time detection system (Bio-Rad) using the following program: 95°C for 15 min and 94°C for 90 s followed by 45 cycles of 94°C for 30 s, 60°C for 30 s, and 72°C for 60 s. See Text S1 in the supplemental material for primer sequences. Relative changes in transcript levels were calculated against the *rpoD* gene (Rru_A2882) as an internal control using the ΔΔ*C_T_* method (58).

### Protein extraction and MS measurements.

Two separate 250-mg aliquots of cell pellet from each 1-liter culture were subjected to detergent-based, heat-assisted cellular lysis for 15 min, as described previously (59). Proteins were extracted and subjected to overnight trypsin (Promega) digestion at 40 µg/mg protein. The resulting peptide solution was desalted, and the solvent was exchanged as previously described (60). Desalted tryptic peptides were loaded onto an in-house-prepared SCX (Luna)-C_18_ (Aqua) resin-packed column (60). For mass spectrometry (MS) analysis, the sample column was connected to a C_18_-packed Picotip (New Objective) interfaced with a Proxeon (Odense, Denmark) nanospray source connected to an LTQ XL mass spectrometer (Thermo Fisher). The mass spectrometer was connected to an UltiMate 3000 HPLC system (Dionex) and operated using Thermo Xcalibur software V2.1.0. Tryptic peptides were eluted and measured via 24-h multidimensional protein identification technology (MuDPIT) coupled with tandem mass spectrometry (2D-LC-MS/MS). Full details may be found in Text S1 in the supplemental material.

### Bioinformatics.

Following MS/MS analysis, raw MS data sets were matched against the *R. rubrum* ATCC 11170 database using the SEQUEST v.27 algorithm set to parameters described elsewhere (60). *R. rubrum* genome sequences were downloaded from JGI’s Integrated Microbial Genomes server (May 2011 version), and common contaminant peptide sequences such as trypsin and keratin were concatenated to the downloaded database. Proteins identified by SEQUEST were further filtered and sorted using DATASelect v.1.9 (61). Predicted cellular localization of *R. rubrum* proteins was obtained via the subcellular localization tool PSORTb version 3.00 (30). Protein-protein interaction analysis was carried out using STRING v. 10.0 (33).

## SUPPLEMENTAL MATERIAL

Text S1  Supplemental materials and methods detailing proteomics procedures as well as strain construction. Download Text S1, PDF file, 0.1 MB

Figure S1  Statistical analysis of proteins identified by deep proteomics. (A) Venn diagram of proteins observed only in sulfate-grown cells, only in MTA-grown cells, and in both. (B) Distribution of identified proteins based on their cellular localization. The cellular localization pattern of expressed proteins in MTA- and sulfate-grown cells was investigated using PSORT v3.0, a subcellular localization prediction tool. Although the number of proteins identified in each treatment differed (SO_4_ fed, 1,197; MTA fed, 1,233), the distribution patterns of proteins per their predicted cellular locations were similar in MTA- and sulfate-grown cells. (C) Functional categories of identified proteins. Number of proteins in sulfate-grown cells (black) and MTA-grown cells (gray) involved in RNA processing and modification (a); chromatin structure and dynamics (b); energy production and conversion (c); cell cycle control, cell division, and chromosome partitioning (d); amino acid transport and metabolism (e); nucleotide transport and metabolism (f); carbohydrate transport and metabolism (g); coenzyme transport and metabolism (h); lipid transport and metabolism (i); translation of ribosomal structure and biogenesis (j); transcription (k); replication, recombination, and repair (l); cell wall membrane and envelope biogenesis (m); cell motility (n); posttranslational modification and protein turnover chaperones (o); inorganic ion transport and metabolism (p); secondary metabolite biosynthesis, transport, and catabolism (q); general function prediction (r); proteins of unknown function (s); signal transduction mechanisms (t); intracellular trafficking, secretion, and vesicular transport (u); and defense mechanisms (v). hyp, hypothetical proteins. Download Figure S1, TIF file, 0.3 MB

Figure S2  STRING analysis of MetINQ homologues. (A) Protein-protein interactions of putative methionine transport proteins in *R. rubrum* were analyzed by STRING v10 analysis using the following proteins as the query: Rru_A0778, lipoprotein YaeC; Rru_A0779, extracellular solute-binding protein; Rru_A0780, binding-protein-dependent transport system inner membrane protein; Rru_A0781, amino acid ABC transporter permease; Rru_A0788, ABC transporter of MetINQ complex; Rru_A0789, binding-protein-dependent transport system inner membrane protein; Rru_A0791, NlpA lipoprotein; Rru_A2417, lipoprotein YaeC; Rru_A2418, binding-protein-dependent transport system inner membrane protein; Rru_A2419, ABC transporter of MetINQ complex. (B) Collapsed view of panel A, showing that *R. rubrum* possesses two putative MetINQ complexes, both of which have components which increase in abundance in response to MTA. Green, protein increased in abundance; black, protein observed but no change in abundance; gray, protein not observed by deep proteomics. Download Figure S2, TIF file, 0.9 MB

Figure S3  Cellular chemotaxis and motility proteins regulated by MTA. Green, protein increased in abundance; black, protein observed but no change in abundance; gray, protein not observed by deep proteomics. Numerous proteins of the rotor complex of the flagellar motor were affected by MTA, including the C-ring (FliG, Rru_A0544, 2-fold; FliN, Rru_A0542, 1.5-fold), which determines rotor rotational direction; the MS-ring (FliF, Rru_A0545, 8.5-fold), which anchors the C-ring to the inner membrane; the proximal rod (FlgC, Rru_A2825, 5-fold), which extends the rotor through the periplasm; and the flagellar hook cap (FlgD, Rru_A2533, 8-fold) protein, which is required for hook assembly. Additionally, the flagellar filament (FliC, Rru_A2858, 1.1-fold), which extends from the hook, also increased in abundance. Stator complex levels (MotA, Rru_A1842/Rru_A1806; MotB, Rru_A1843/Rru_A1807) of the flagellar motor, which drives flagellar rotation and regulates rotation speed, are not altered by MTA. The asterisk denotes the CheB Tn*5* mutant (ΔRru_A1406), which was incapable of growth on MTA (Fig. 5). Download Figure S3, TIF file, 0.5 MB

Figure S4  Identification of purine salvage nucleosides and bases. (A) HPLC analysis at 260-nm detection of known standards. (B) HPLC analysis of purines produced by *R. rubrum* wild-type strain after 120 min post-anaerobic feeding with MTA before treatment with xanthine oxidase (− Xan Ox) and after treatment with xanthine oxidase (+ Xan Ox). The hypoxanthine peak present before treatment with xanthine oxidase is converted by xanthine oxidase to urate upon treatment, confirming the presence of hypoxanthine. *R_T_*, retention time, in minutes. (C) Mass spectrometry of major species present in panel B with retention times between 20 and 22 min. The only purine identified was adenine. No inosine or xanthosine was observed. Download Figure S4, TIF file, 0.2 MB

Figure S5  Metabolite identification by radiochromatography. (A) Reversed-phase separation and ^14^C radiometric detection of MTA metabolism standards. Bottom trace, [*methyl*-^14^C]MTA treated with MTA phosphorylase (Rru_A0361) to produce MTR-1P (peak 1). Middle trace, MTR-1P treated with MTR-1P isomerase (Rru_A0360) to produce MTRu-1P (peak 2). Top trace, MTRu-1P treated with MTRu-1P isomerase (Rru_A1998) to produce a 3:1 mixture of MTXu-1P (peak 3) and MTRu-5P (peak 4). (B) Reversed-phase separation and ^14^C radiochromatography detection of *R. rubrum* MTA phosphorylase deletion strain (ΔRru_A0361) grown anaerobically on sulfate and then fed with [*methyl*-^14^C]MTA for the indicated time (minutes) before resolution of metabolites. *R_T_*, retention time. No metabolism of MTA was observed (MTA peak). The methylthioribose (MTR) and unknown (Unk) peaks are due to trace amounts of impurity from the manufacturer present in the initial MTA sample fed to the cells. Download Figure S5, TIF file, 0.2 MB

Figure S6  Potential anaerobic MTA metabolism in other organisms. BLASTp alignment of *R. rubrum* MTXu-5P sulfurylase (1, Rru_A2000, cupin) and MTRu-1P isomerase (2, Rru_A1998, RLP) visualized using Seed Viewer 2.0. Download Figure S6, TIF file, 0.1 MB

Table S1  Proteins observed to change in abundance level in response to MTA. NSAF is the normalized spectral abundance factor. Av. Adj. NSAF is the average of the NSAFs from the 4 technical replicates for MTA- and sulfate-fed cells. Table S1, XLSX file, 1.7 MB

Table S2  All proteins observed by deep proteomics. NSAF is the normalized spectral abundance factor. Av. Adj. NSAF is the average of the NSAFs from the 4 technical replicates for MTA- and sulfate-fed cells. PI, isoelectric point; MW, molecular weight; COG, clusters of orthologous groups. Table S2, XLSX file, 0.3 MB
