# Supplementary material for: Metabolic Regulation as a Consequence of Anaerobic 5-Methylthioadenosine Recycling in Rhodospirillum rubrum
Source: mBio. 2016 Jul 12;7(4):e00855-16. doi: 10.1128/mBio.00855-16 (PMC4958253; doi:10.1128/mBio.00855-16)
Supplement: Text S1 — Supplemental materials and methods detailing proteomics procedures as well as strain construction. Download [file mbo003162887s1.pdf]

Supplemental Methods

Cellular lysis and protein extraction: Two separate 250 mg aliquots of cell pellet from each 1 liter culture (two with sulfate and two with MTA as sole sulfur source) was subjected to detergent based–heat assisted cellular lysis for 15 minutes as described previously (1). Following centrifugation at 21,000 g for 15 min, the supernatant was chilled and TCA added to 25% final concentration. The solution was incubated overnight at -20 °C and centrifuged at 21,000 g. The resulting protein pellet was washed twice with chilled acetone, air dried, and re-suspended in guanidine buffer [6M guanidine HCl in Tris CaCl2 buffer, pH 8.5 (50mM Tris, 10mM $CaCl_2$)] and incubated at 60 °C for 4 h. An aliquot of 25 µl was removed for protein estimation using the RC/DC protein estimation kit (Bio-Rad) as per the manufacturer's protocol. The remainder of the protein mixture was diluted six-fold with Tris-$CaCl_2$ buffer and subjected to overnight trypsin (Promega) digest at 40 µg trypsin per 1 mg protein. The resulting peptide solution was desalted and the solvent exchanged as described earlier (2).

NanoLC-MS/MS Analysis - Desalted tryptic peptides were loaded onto an in-house prepared SCX (Luna)-C18 (Aqua) resin packed column (2) and subjected to an offline wash with solvent A (5% acetonitrile, 0.1% formic acid in HPLC-grade water) for 5 min followed by a gradient towards 100% solvent B (70% acetonitrile, 0.1% formic acid in HPLC-grade water) over 10 min. For MS analysis, the sample column was connected to a C18-packed Picotip (New Objective) interfaced with a Proxeon (Odense) nanospray source connected to an LTQ XL mass spectrometer (Thermo Fisher). The mass spectrometer was connected to an Ultimate 3000 HPLC system (Dionex) and operated using Thermo Xcalibur software V2.1.0. Tryptic peptides were

eluted and measured via 24-h Multi-Dimensional Protein Identification Technology (MuDPIT) coupled with tandem mass spectrometry (2D-LC-MS/MS). The mass spectrometer was operated in a data dependent mode using the following parameters: collision-activated dissociation of five parent ions was carried out after each full scan. Two microscans were averaged for every full MS and MS/MS spectrum and a 3 m/z isolation width was applied. Fragmentation was achieved using 35% collision energy with a dynamic exclusion repeat of 1 with duration of 60 seconds.

Bioinformatics and Data Analysis - Following MS/MS analysis, raw MS datasets were matched against *R. rubrum* ATCC 11170 database using SEQUEST v.27 algorithm set to parameters described elsewhere (2). *R. rubrum* genome sequences were downloaded from JGI's Integrated Microbial Genomes server (May 2011 version) and common contaminant peptide sequences such as trypsin and keratin were concatenated to the downloaded database. Proteins identified by SEQUEST were further filtered and sorted using DTASelect v. 1.9 (3) using the following parameters: fully tryptic peptides only with ΔCN of at least 0.08 and cross-correlation scores (Xcorr) of at least 1.8 (+1), 2.5 (+2), and 3.5 (+3). Identification of at least two peptides for a given protein sequence was set as a prerequisite for protein identification. All the proteins identified in MTA-fed and sulfate fed cells are listed in Supplemental Table 2 and raw datasets and databases will be available upon request. Spectral counts for identified proteins were normalized as described earlier (4), using a Poisson regression model (5), *p* values generated, which were further adjusted using the Benjamini and Hochberg correction (6) to obtain the *q* value and a cut off value of $q < 0.05$ (corresponding to 1% FDR) was used to select for proteins that were differentially expressed between the two groups under comparison.

Knockout strains - The MTA phosphorylase gene (Rru_A0361) was deleted by homologous recombination using the SacB selection system. *R. rubrum* genomic DNA fragment 0361A was amplified by PCR using primers 361KF (CGAGGAGCCCCTCGAGACCATCACCCAGCTCGC) and 361XR (CCCGATCCGCCGATTCTAGAAAGCACCGGCTGC) and fragment 0361B was amplified using primers 361SF (GGATGGCGCGCAAAACTAGTGCCGTCGCCGGTCGG) and 361KR (GCAAACGCCGCATGCGGTGATCAAGCCGGTGATCA). XbaI and SpeI (New England Biolabs) digested 0361A and 0361B fragments were ligated together using T4 Ligase (New England Biolobs) and agarose gel purified (Qiagen) to produce a truncated Rru_0361 gene fragment consisting of the first and last 10 amino acid residues and 1000 bp upstream and downstream of the truncated gene. SphI and XhoI digested gene fragment was cloned into SphI and SalI digested pK18-mobsacB suicide vector (ATCC87097), transformed into *E. coli* S17 and conjugated into *R. rubrum* via biparental mating. Knockout strains were selected by sucrose sensitivity and verified by sequencing the deleted genomic region.

The MTR-1P isomerase gene (Rru_A0360) was similarly deleted by homologous recombination using the SacB selection system. R. *rubrum* genomic DNA fragment Ru_360 was amplified by PCR using primers 360KF (GGGGAACATATGTCCGAGGCGTATCGGC) and 360KR (GCGACCGCGGATCCGGTCGGGAAACGAGGCG) and cloned into pTOPO-Blunt (Life Technologies) to create pTOPO-R360. pTOPO-R360 was digested with EcoRI and the insert was ligated into EcoRI digested pK18-mobsacB to create plasmid pK18-R360. The isomerase gene was disrupted by cloning in a gentamycin antibiotic resistance cassette into the genes native NheI site. The cassette was amplified from pUC1318-gm (7) using primers GenF (CGGTTCGTAAACTGTAATGCTAGCAGCGTATGCGCTCAC) and GenR

(CGCTGCAAGAAGGCTAGCTGGTGGCGCTTGC), digested with NheI and ligated using T4 ligase into NheI digested pK18-R360 to create plasmid pK18-R360gm. This plasmid was transformed into *E. coli* SM10 and conjugated into *R. rubrum* via biparental mating. Knockout strains were selected by sucrose sensitivity and verified by sequencing the deleted genomic region.

Primers for RT-qPCR

MTAP:	Ru0361-F	AGCAATCTCTATCGGCAATG
	Ru0361-R	AGCAGTCGAAATCGGTGAC
RLP:	Ru1998-F	ATCGATACCTTCCTGATCGC
	Ru1998-R	GAACAGCTTGCCGAAATG
cbbM:	Ru2400-F	TATACCGCCTTCGTCCATTGC
	Ru2400-R	CGTCCTGGGTCAGCATATAG
Phasin:	Ru2817-F	CAAGTCGTTCGACAGCGT
	Ru2187-R	GTCGACATCTCGGAAACCTTGC
RecA:	Ru2819-F	TGAAGGACAAGGAGGAGGTC
	Ru2819-R	CGCCATACATGATGTCGAA
RpoD:	Ru2822-F	AGCTTGTCGTATTTGCGCTC
	Ru2822-R	ACCTTCGAGCATATCGCCAC

Supplemental References

1. (53) **Schmittgen TD, Livak KJ.** 2008. Analyzing real-time PCR data by the comparative C(T) method. Nat Protoc. 3:1101-8.
2. (54) **Chourey K, Jansson J, VerBerkmoes N, Shah M, Chavarria KL, Tom LM, Brodie EL, Hettich RL.** 2010. Direct cellular lysis/protein extraction protocol for soil metaproteomics. J Proteome Res. 9:6615-22.

3. **Sharma R, Dill BD, Chourey K, Shah M, VerBerkmoes NC, Hettich RL.** 2012. Coupling a detergent lysis/cleanup methodology with intact protein fractionation for enhanced proteome characterization. J Proteome Res. 11:6008-18.
4. **Chourey K, Thompson MR, Shah M, Zhang B, Verberkmoes NC, Thompson DK, Hettich RL**. 2009. Comparative temporal proteomics of a response regulator (SO2426)-deficient strain and wild-type *Shewanella oneidensis* MR-1 during chromate transformation. J. Proteome Res. 8, 59–71.
5. **Dobson A J**. 2002. An Introduction to Generalized Linear Models. Chapman & Hall CRC, London, England.
6. **Benjamini Y and Hochberg Y.** 1995. Controlling the false discovery rate: a practical and powerful approach to multiple testing. J. R. Statist. Soc. B. 57, 289–300.
7. **Arsene F, Kaminski PA, Elmerich C**. 1996. Modulation of NifA activity by PII in Azospirillum brasilense: evidence for a regulatory role of the NifA N-terminal domain. J. Bacteriol. 178, 4830-4838.

Yu NY, Wagner JR, Laird MR, Melli G, Rey S, Lo R, Dao P, Sahinalp SC, Ester M, Foster LJ, Brinkman FS. 2012. PSORTb 3.0: improved protein subcellular localization prediction with refined localization subcategories and predictive capabilities for all prokaryotes. Bioinformatics. 26:1608-15.
