# Supplementary figures and images for: Metabolic Regulation as a Consequence of Anaerobic 5-Methylthioadenosine Recycling in Rhodospirillum rubrum
Source: mBio. 2016 Jul 12;7(4):e00855-16. doi: 10.1128/mBio.00855-16 (PMC4958253; doi:10.1128/mBio.00855-16)

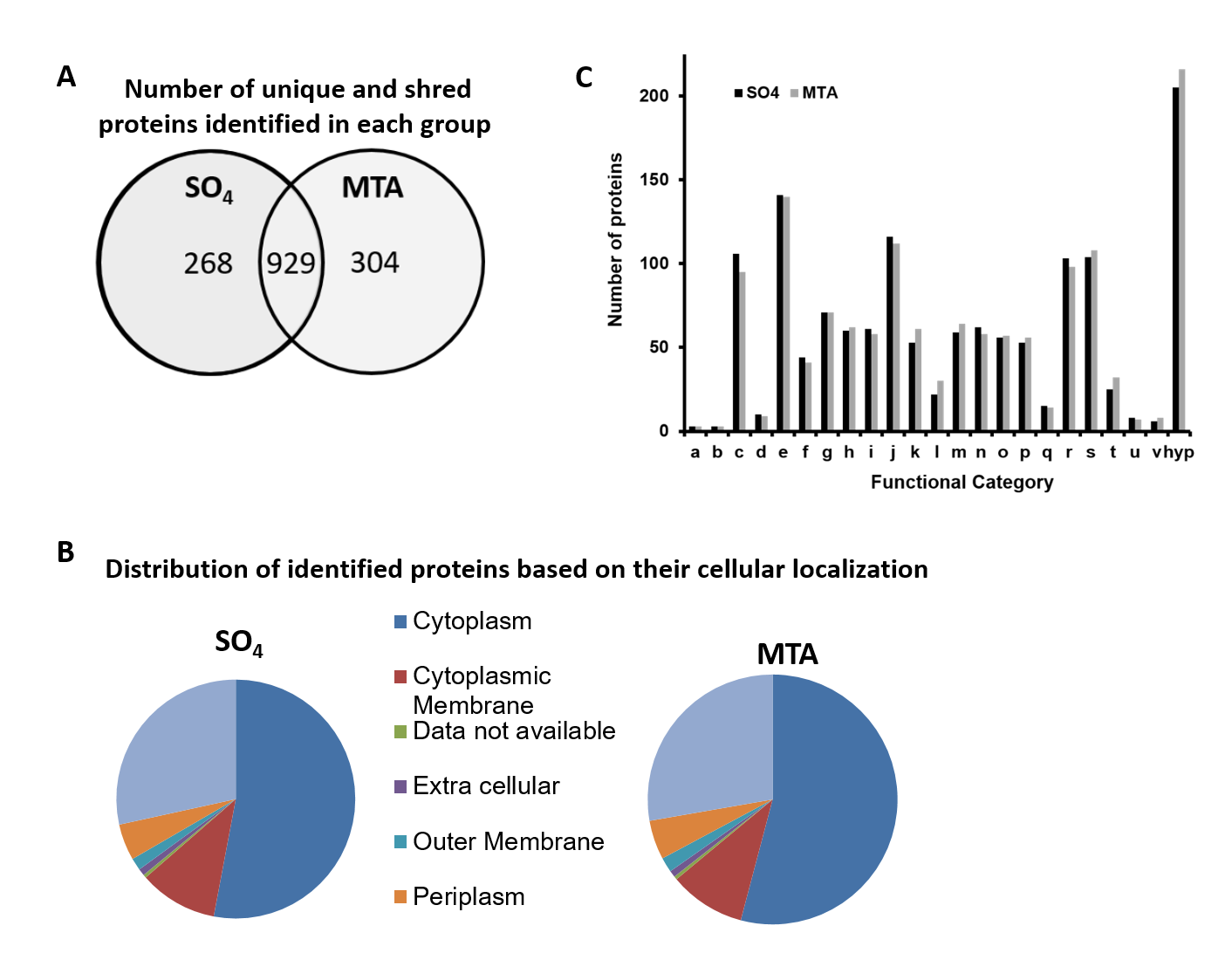

Supplement: Figure S1 — Statistical analysis of proteins identified by deep proteomics. (A) Venn diagram of proteins observed only in sulfate-grown cells, only in MTA-grown cells, and in both. (B) Distribution of identified proteins based on their cellular localization. The cellular localization pattern of expressed proteins in MTA- and sulfate-grown cells was investigated using PSORT v3.0, a subcellular localization prediction tool. Although the number of proteins identified in each treatment differed (SO4 fed, 1,197; MTA fed, 1,233), the distribution patterns of proteins per their predicted cellular locations were similar in MTA- and sulfate-grown cells. (C) Functional categories of identified proteins. Number of proteins in sulfate-grown cells (black) and MTA-grown cells (gray) involved in RNA processing and modification (a); chromatin structure and dynamics (b); energy production and conversion (c); cell cycle control, cell division, and chromosome partitioning (d); amino acid transport and metabolism (e); nucleotide transport and metabolism (f); carbohydrate transport and metabolism (g); coenzyme transport and metabolism (h); lipid transport and metabolism (i); translation of ribosomal structure and biogenesis (j); transcription (k); replication, recombination, and repair (l); cell wall membrane and envelope biogenesis (m); cell motility (n); posttranslational modification and protein turnover chaperones (o); inorganic ion transport and metabolism (p); secondary metabolite biosynthesis, transport, and catabolism (q); general function prediction (r); proteins of unknown function (s); signal transduction mechanisms (t); intracellular trafficking, secretion, and vesicular transport (u); and defense mechanisms (v). hyp, hypothetical proteins. Download [file mbo003162887sf1.tif]

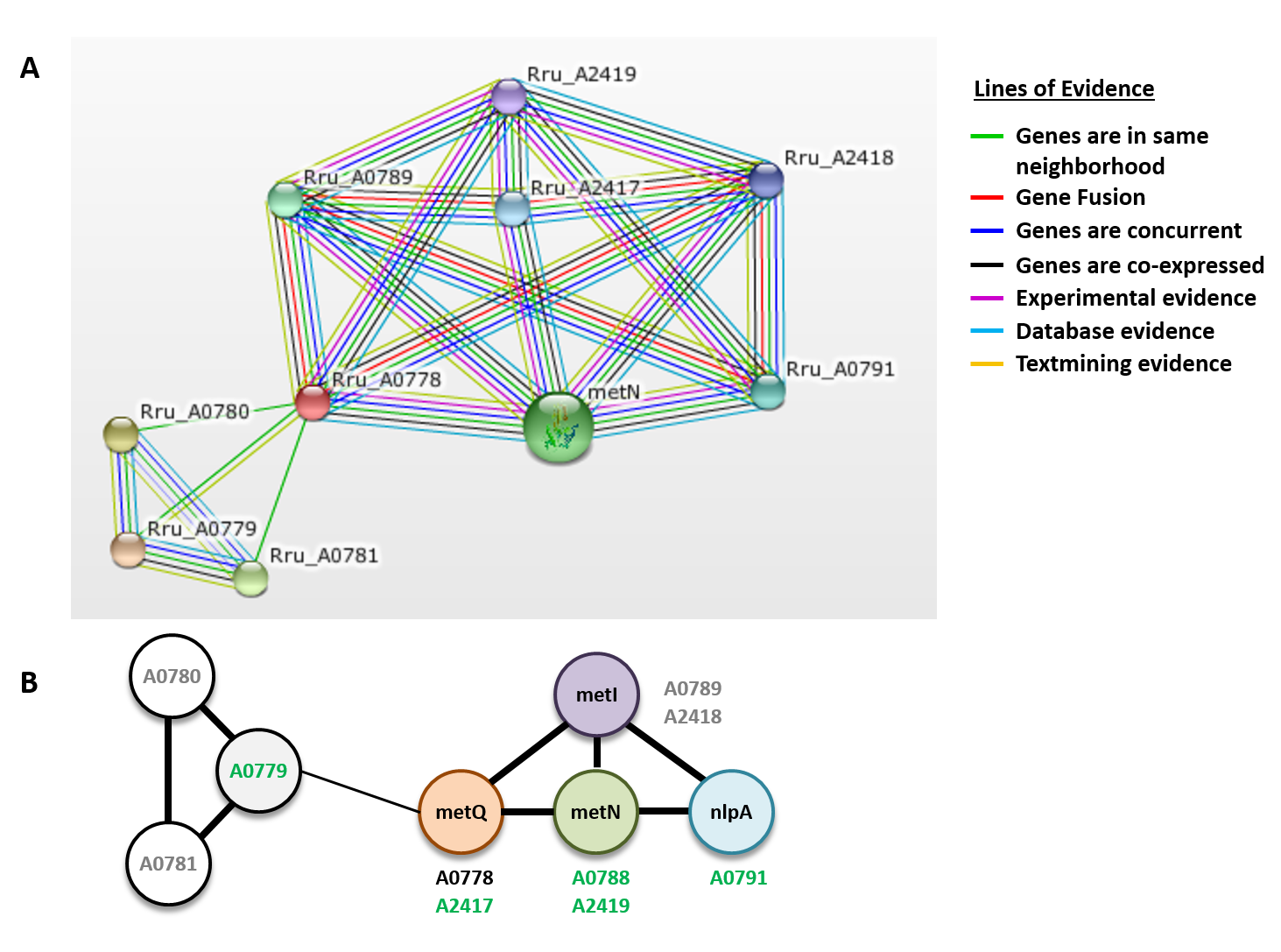

Supplement: Figure S2 — STRING analysis of MetINQ homologues. (A) Protein-protein interactions of putative methionine transport proteins in R. rubrum were analyzed by STRING v10 analysis using the following proteins as the query: Rru_A0778, lipoprotein YaeC; Rru_A0779, extracellular solute-binding protein; Rru_A0780, binding-protein-dependent transport system inner membrane protein; Rru_A0781, amino acid ABC transporter permease; Rru_A0788, ABC transporter of MetINQ complex; Rru_A0789, binding-protein-dependent transport system inner membrane protein; Rru_A0791, NlpA lipoprotein; Rru_A2417, lipoprotein YaeC; Rru_A2418, binding-protein-dependent transport system inner membrane protein; Rru_A2419, ABC transporter of MetINQ complex. (B) Collapsed view of panel A, showing that R. rubrum possesses two putative MetINQ complexes, both of which have components which increase in abundance in response to MTA. Green, protein increased in abundance; black, protein observed but no change in abundance; gray, protein not observed by deep proteomics. Download [file mbo003162887sf2.tif]

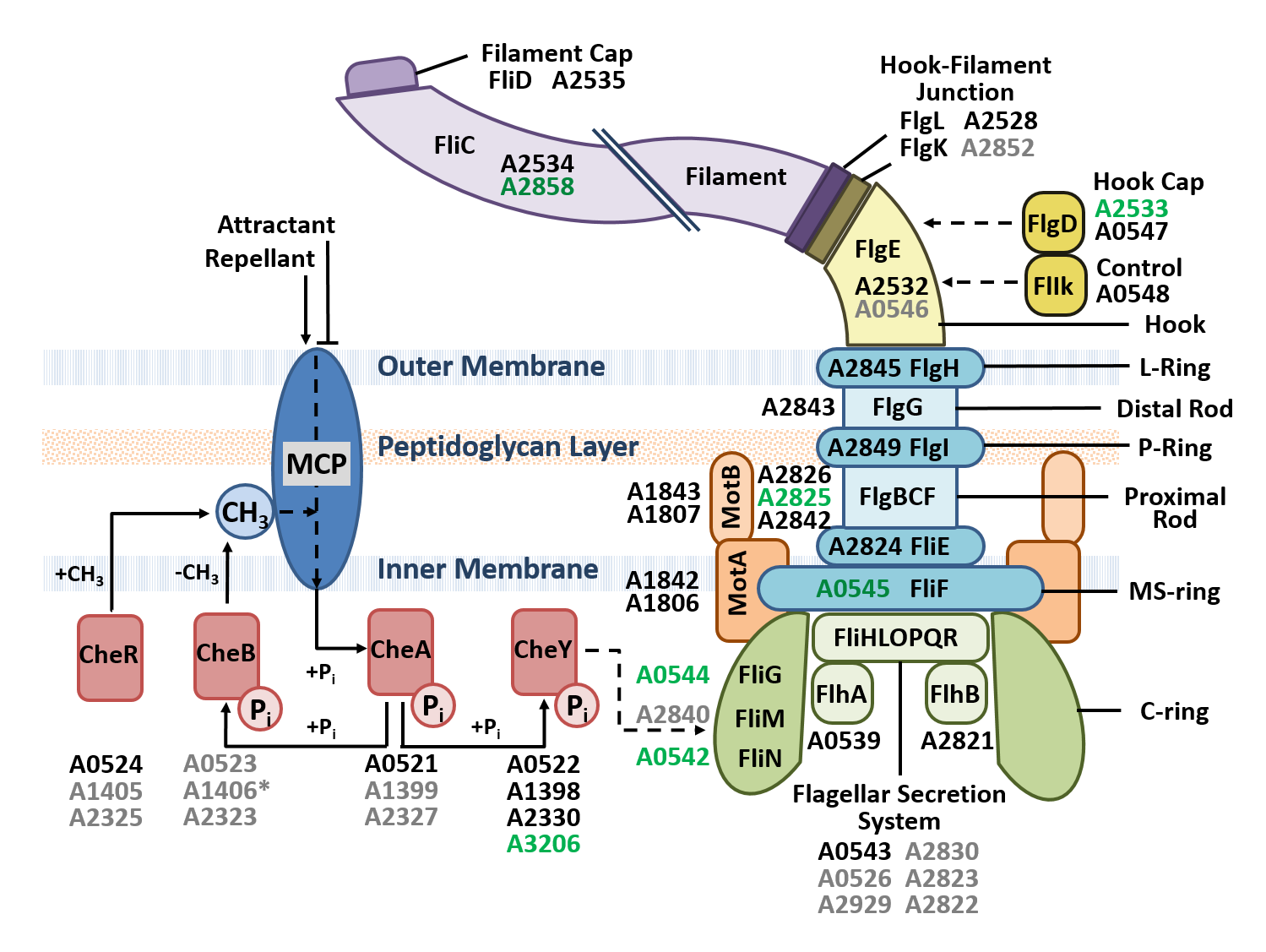

Supplement: Figure S3 — Cellular chemotaxis and motility proteins regulated by MTA. Green, protein increased in abundance; black, protein observed but no change in abundance; gray, protein not observed by deep proteomics. Numerous proteins of the rotor complex of the flagellar motor were affected by MTA, including the C-ring (FliG, Rru_A0544, 2-fold; FliN, Rru_A0542, 1.5-fold), which determines rotor rotational direction; the MS-ring (FliF, Rru_A0545, 8.5-fold), which anchors the C-ring to the inner membrane; the proximal rod (FlgC, Rru_A2825, 5-fold), which extends the rotor through the periplasm; and the flagellar hook cap (FlgD, Rru_A2533, 8-fold) protein, which is required for hook assembly. Additionally, the flagellar filament (FliC, Rru_A2858, 1.1-fold), which extends from the hook, also increased in abundance. Stator complex levels (MotA, Rru_A1842/Rru_A1806; MotB, Rru_A1843/Rru_A1807) of the flagellar motor, which drives flagellar rotation and regulates rotation speed, are not altered by MTA. The asterisk denotes the CheB Tn5 mutant (ΔRru_A1406), which was incapable of growth on MTA (Fig. 5). Download [file mbo003162887sf3.tif]

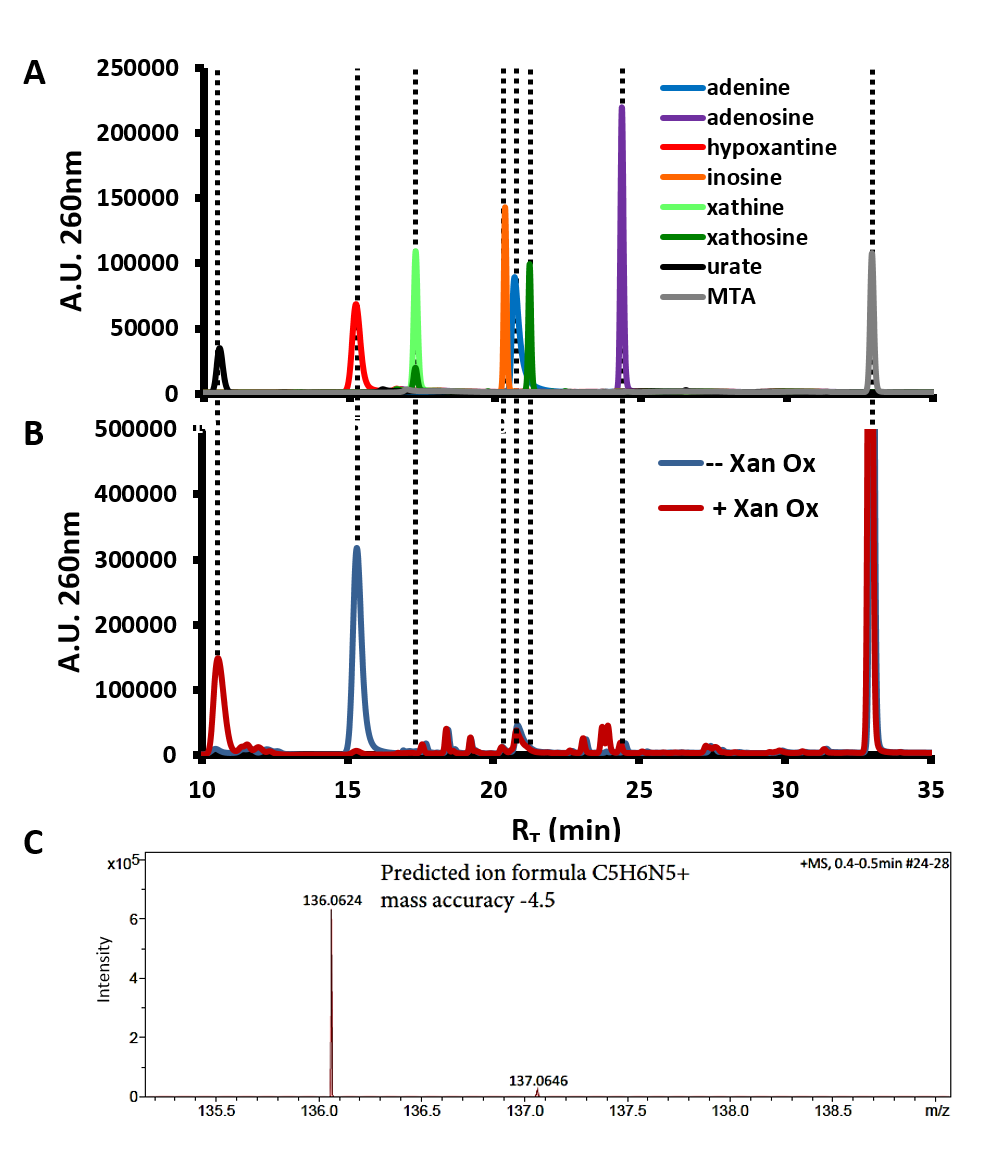

Supplement: Figure S4 — Identification of purine salvage nucleosides and bases. (A) HPLC analysis at 260-nm detection of known standards. (B) HPLC analysis of purines produced by R. rubrum wild-type strain after 120 min post-anaerobic feeding with MTA before treatment with xanthine oxidase (− Xan Ox) and after treatment with xanthine oxidase (+ Xan Ox). The hypoxanthine peak present before treatment with xanthine oxidase is converted by xanthine oxidase to urate upon treatment, confirming the presence of hypoxanthine. RT, retention time, in minutes. (C) Mass spectrometry of major species present in panel B with retention times between 20 and 22 min. The only purine identified was adenine. No inosine or xanthosine was observed. Download [file mbo003162887sf4.tif]

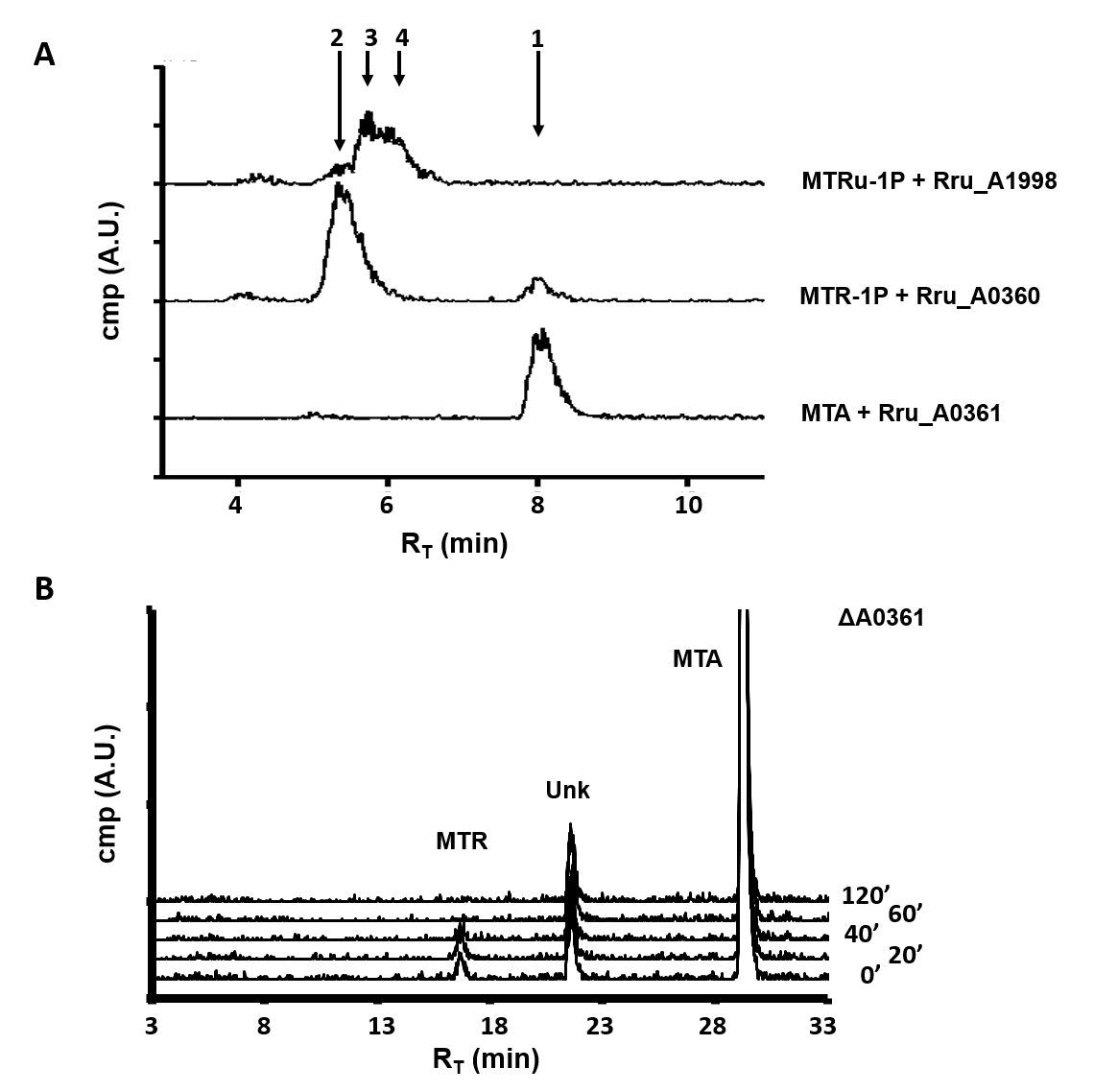

Supplement: Figure S5 — Metabolite identification by radiochromatography. (A) Reversed-phase separation and 14C radiometric detection of MTA metabolism standards. Bottom trace, [methyl-14C]MTA treated with MTA phosphorylase (Rru_A0361) to produce MTR-1P (peak 1). Middle trace, MTR-1P treated with MTR-1P isomerase (Rru_A0360) to produce MTRu-1P (peak 2). Top trace, MTRu-1P treated with MTRu-1P isomerase (Rru_A1998) to produce a 3:1 mixture of MTXu-1P (peak 3) and MTRu-5P (peak 4). (B) Reversed-phase separation and 14C radiochromatography detection of R. rubrum MTA phosphorylase deletion strain (ΔRru_A0361) grown anaerobically on sulfate and then fed with [methyl-14C]MTA for the indicated time (minutes) before resolution of metabolites. RT, retention time. No metabolism of MTA was observed (MTA peak). The methylthioribose (MTR) and unknown (Unk) peaks are due to trace amounts of impurity from the manufacturer present in the initial MTA sample fed to the cells. Download [file mbo003162887sf5.tif]

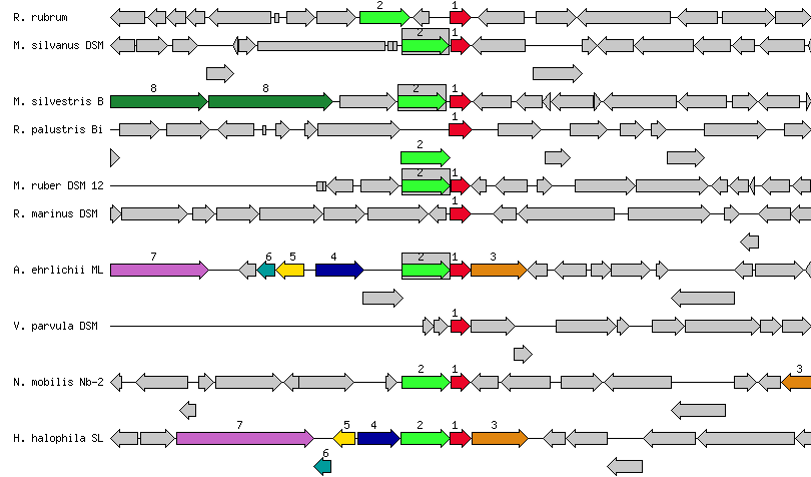

Supplement: Figure S6 — Potential anaerobic MTA metabolism in other organisms. BLASTp alignment of R. rubrum MTXu-5P sulfurylase (1, Rru_A2000, cupin) and MTRu-1P isomerase (2, Rru_A1998, RLP) visualized using Seed Viewer 2.0. Download [file mbo003162887sf6.tif]
